# Altered cytoskeleton dynamics in patient-derived iPSC-based model of PCDH19 clustering epilepsy

**DOI:** 10.3389/fcell.2024.1518533

**Published:** 2025-01-06

**Authors:** Rossella Borghi, Stefania Petrini, Valentina Apollonio, Marina Trivisano, Nicola Specchio, Sandra Moreno, Enrico Bertini, Marco Tartaglia, Claudia Compagnucci

**Affiliations:** ^1^ Molecular Genetics and Functional Genomics, Bambino Gesù Children’s Hospital, IRCCS, Rome, Italy; ^2^ Confocal Microscopy Core Facility, Laboratories, Bambino Gesù, Children’s Research Hospital, IRCCS, Rome, Italy; ^3^ Neurology, Epilepsy and Movement Disorders Unit, Bambino Gesù Children’s Hospital, IRCCS, Full Member of European Reference Network EpiCARE, Rome, Italy; ^4^ Department of Science, LIME, University Roma Tre, Rome, Italy; ^5^ Research Unit of Neuromuscular and Neurodegenerative Disorders, Bambino Gesù Children’s Hospital, IRCCS, Rome, Italy

**Keywords:** PCDH19, epilepsy, IPSC-derived neurons, microfilaments, microtubules, cytoskeletal dynamics, neurodevelopment

## Abstract

Protocadherin 19 (PCDH19) is an adhesion molecule involved in cell-cell interaction whose mutations cause a drug-resistant form of epilepsy, named PCDH19-Clustering Epilepsy (PCDH19-CE, MIM 300088). The mechanism by which altered PCDH19 function drive pathogenesis is not yet fully understood. Our previous work showed that PCDH19 dysfunction is associated with altered orientation of the mitotic spindle and accelerated neurogenesis, suggesting a contribution of altered cytoskeleton organization in PCDH19-CE pathogenesis in the control of cell division and differentiation. Here, we evaluate the consequences of altered PCDH19 function on microfilaments and microtubules organization, using a disease model obtained from patient-derived induced pluripotent stem cells. We show that iPSC-derived cortical neurons are characterized by altered cytoskeletal dynamics, suggesting that this protocadherin has a role in modulating stability of MFs and MTs. Consistently, the levels of acetylated-tubulin, which is related with stable MTs, are significantly increased in cortical neurons derived from the patient’s iPSCs compared to control cells, supporting the idea that the altered dynamics of the MTs depends on their increased stability. Finally, performing live-imaging experiments using fluorescence recovery after photobleaching and by monitoring GFP-tagged end binding protein 3 (EB3) “comets,” we observe an impairment of the plus-end polymerization speed in PCDH19-mutated cortical neurons, therefore confirming the impaired MT dynamics. In addition to altering the mitotic spindle formation, the present data unveil that PCDH19 dysfunction leads to altered cytoskeletal rearrangement, providing therapeutic targets and pharmacological options to treat this disorder.

## 1 Introduction

Protocadherin 19 (PCDH19) is a calcium-dependent cell-cell adhesion glycoprotein belonging to the group of δ2 non-clustered protocadherins (Dibbens et al., 2008). PCDH19 pathogenic variants cause a rare disease characterized by developmental and epileptic encephalopathy (DEE9) known as PCDH19-Clustering Epilepsy (PCDH19-CE) (Dibbens et al., 2008). PCDH19-CE mainly affects females, with early onset seizures, initially associated with fever, which usually occurs in clusters. Additionally, autistic and psychiatric features have been reported in some patients (Hynes et al., 2010; Jamal et al., 2010; Marini et al., 2010; Specchio et al., 2011; Piton et al., 2011; Breuillard et al., 2016). This genetic disease is caused by a number of variants (more than 175; Niazi et al., 2019) or a partial deletion of the *PCDH19* gene, localized on the long arm of chromosome X (Xq22.3). PCDH19-CE is characterized by an unusual pattern of inheritance. Disorders caused by mutations on the X chromosome are typically characterized by affected males and unaffected carrier females. In contrast, this disease affects heterozygous females, leaving transmitting males (seemingly) unaffected and phenotypically normal. This mechanism causes PCDH19 to have a unique mode of transmission and it can be explained by “cellular interference model,” which is defined as the coexistence of mutated PCDH19 and wild-type neurons that do not allow the two cell populations to properly interact (Depienne et al., 2009).

As other protocadherins, PCDH19 is predominantly expressed in the developing and adult nervous system of the vertebrates with specific spatial and temporal expression patterns (Emond et al., 2009; Biswas et al., 2010; Kim et al., 2011; Hirano and Takeichi, 2012; Pederick et al., 2016; Pederick et al., 2018; Pancho et al., 2020; Tsai et al., 2020), suggesting that it plays a relevant role in neurodevelopmental processes (e.g., neuronal differentiation, axon guidance, dendritic arborization and self-avoidance, synapse formation and dynamics). In fact, several studies reported that PCDH19 dysfunction leads to increased neuronal development (Cooper et al., 2015; Fujitani et al., 2017; Homan et al., 2018; Borghi et al., 2021), increased neurites’ length (Homan et al., 2018; Borghi et al., 2021; Mincheva-Tasheva et al., 2021), impaired neuronal migration (Biswas et al., 2010; Pederick et al., 2016; Lv et al., 2019; Bassani et al., 2018; Hoshina et al., 2021; Cwetsch et al., 2022), and abnormal cell sorting during neuronal differentiation (Pederick et al., 2016; Hayashi et al., 2017; Niu et al., 2024). PCDH19 function has also been associated to synaptic transmission and formation of synaptic connections during brain development (Bassani et al., 2018; Mincheva-Tasheva et al., 2021); however, the mechanism by which altered PCDH19 function causes PCDH19-CE is still poorly characterized. As a consequence, no efficacious treatment is available for patients.

Since neuronal development is a process that depends on the balance between cytoskeleton dynamics and stability (Yokota et al., 2007; Sakakibara et al., 2013; Compagnucci et al., 2016a; Lee et al., 2017), we hypothesized that PCDH19 has a role in cytoskeletal organization and function. This hypothesis is supported by a recent study providing evidence that PCDH19 is relevant in cytoskeletal functionality, since its interactome is enriched in actin and tubulin associated proteins and in regulators of cell division (Emond et al., 2021). In particular, a role for PCDH19 in MF dynamics has strongly been suggested by the evidence of its interaction with the WAVE (WASP family verprolin homologous protein) regulatory complex (WRC) (Tai et al., 2010; Chen et al., 2014; Hayashi et al., 2017), through which it may regulate actin filaments polymerization and ramification. A role in MT organization and function is supported by the demonstration that PCDH19 localized at centrosome, and its dysfunction leads to altered mitotic spindle morphology and orientation (Borghi et al., 2021). Based on these considerations, we aimed to investigate the impact of altered PCDH19 function on cytoskeletal organization using an *in vitro* model based on patient-derived iPSCs to obtain human cortical neurons.

## 2 Materials and methods

### 2.1 iPSCs maintenance and differentiation into cortical neurons

Human control (Ctrl) iPSC lines were purchased from System Biosciences, obtained from skin fibroblasts of an adult healthy male and reprogrammed using non-integrating episomal technology.

Mutated iPSCs were obtained from a mosaic male patient carrying the pathogenic variant c.1352C > T, p.Pro451Leu on the *PCDH19* gene, as described in Borghi et al. (2021) and they were maintained in culture in six well plates, pre-coated with Matrigel (354277, Corning), with mTeSR plus medium (100-0276, StemCell Technologies) at 37°C, 5% O_2_. iPSCs were differentiated into cortical neurons following the protocol described in Borghi et al. (2021).

### 2.2 Immunofluorescence assay

Cells were grown on microscopy glass slides pre-coated with Matrigel and then fixed with 4% paraformaldehyde (PFA) for 10 min at RT, washed twice with PBS, blocked and permeabilized with a solution containing 5% bovine serum albumin (BSA, Sigma-Aldrich) and 0.1% Triton X-100 (Sigma-Aldrich) for 1 h at RT. Primary antibodies included anti-βIII-tubulin (1:500, 2 h at RT, T8578 Sigma), anti-β actin (1:200, ON at 4°C, A2228 Sigma-Aldrich), anti-acetylated tubulin (1:200, 2 h at RT, T7451 Sigma-Aldrich), anti-STOP (stable-tubule-only-polypeptide or microtubule-associated protein 6 - MAP6) (1:100, ON at 4°C, sc-53513 Santacruz). Secondary antibodies were conjugated with anti-mouse or anti-rabbit Alexafluor 488 or 555 (Invitrogen). Coverslips were mounted using PBS/Glycerol (1:1), visualized using a laser-scanning confocal microscope Leica TCS-SP8X (Leica Microsystems) and acquired using the LASX software.

### 2.3 Actin polymerization assay

**FIGURE 1:**
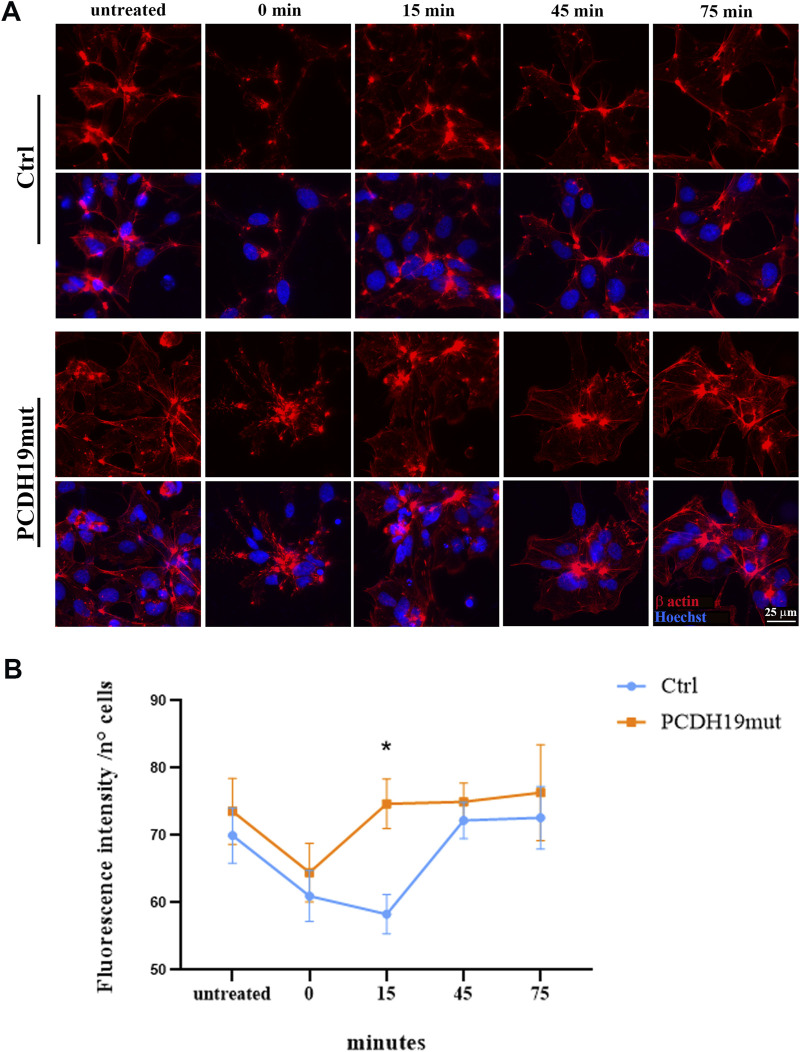
Assessment of microfilaments dynamics in PCDH19mut iPSC-derived cortical neurons. MFs re-polymerization in control (Ctrl) and PCDH19mut cortical neurons following cytochalasin D treatment and washout. **(A)** Representative images of the cells stained with anti-β-actin antibody (red) and Hoechst33342 (blue) are depicted: before the cytochalasin D treatment (untreated), at 0, 15, 45, and 75 min following cytochalasin D washout (0, 15, 45, 75 min). Scale bar = 25 μm. **(B)** Quantification of β-actin fluorescent signal intensity. A minimum of n = 3 and 9 images were quantized per experimental condition. Data are represented as mean ± SEM of three independent experiments and the number of cells analysed is between 901 and 997 per experimental condition. Significance was tested using ordinary two-way ANOVA (parametric tests). *p ≤ 0.05.

At the end of neuronal differentiation, the iPSC-derived cortical neurons were grown on microscopy glasses (with a diameter of 10 mm) pre-coated with Matrigel to perform MF polymerization assays. Depolymerization of MFs was induced by adding cytochalasin D (1 μΜ for 60 min at 37°C, C2618 Sigma-Aldrich) and the cells were fixed using 4% PFA before the treatment (indicated as untreated in Figure 1), soon after the treatment and cytochalasin D washout (indicated as time 0 min in Figure 1), and 15, 45 and 75 min following cytochalasin D washout (15, 45, 75 min, respectively). The cells were immunostained with β-actin and nuclei counterstained with Hoechst33342 (Cod H3570, Invitrogen) and their images acquired using a HC PL APO 40x/0.85 DRY objective of an inverted Leica DMi8 microscope (exposure time 136.855 ms - emission wavelength 460 nm; exposure time 402.023 ms - emission wavelength 585 nm, Leica Microsystems) with a Hamamatsu-C11440-42U-USB-101662 camera and are representative of three experiments. The fluorescent intensity signal of the β-actin has been quantified using Fiji software (Image J) and normalized to the number of cells (by manual counting) at the different time points. The obtained data were graphed using GraphPad Prism software.

### 2.4 Microtubule polymerization assay

Following neuronal differentiation, the iPSC-derived cortical neurons were grown (as for actin polymerization assays) on microscopy glasses (with a diameter of 10 mm) pre-coated with Matrigel to perform MT polymerization assays. Depolymerization of MTs was obtained by adding nocodazole (10 μΜ, M1404 Sigma-Aldrich) for 30 min at 37°C. Cells were fixed in 4% PFA at different time points (before the treatment and after 0, 15, 45, 75 min from Nocodazole washout) and immunostained with βIII-tubulin to mark MTs. Nuclei are counterstained with Hoechst33342 (Cod H3570, Invitrogen). Images were acquired using a HC PL APO 40x/0.85 DRY objective of an inverted Leica DMi8 microscope (exposure time 92.521 ms - emission wavelength 460 nm; exposure time 220.020 ms - emission wavelength 585 nm, Leica Microsystems) with a Hamamatsu-C11440-42U-USB-101662 camera and are representative of three experiments. The fluorescent intensity signal of the βΙΙΙ-tubulin at the different time points has been quantified using Fiji software (Image J) and normalized to the number of cells (by manual counting). The obtained data were graphed using GraphPad Prism software.

### 2.5 Photobleaching experiments

Cortical neurons were stained with 1 μΜ fluorescent probe SiR-tubulin (CY-SC002, Spirochrome) to perform live imaging experiments to analyse the α-tubulin dynamics using photobleaching technique. Confocal time-lapse microscopy of the fluorescence recovery after photobleaching (FRAP) experiments were performed on a Leica TCS-SP8X AOBS laser-scanning confocal microscope (Leica Microsystems, Mannheim, Germany), equipped with a white light laser (470–670 nm) source, a 405 nm laser, and an environment box (OkoLab) allowing to maintain stable conditions of temperature, CO_2_ and humidity during live cell imaging. Images were acquired using a PlanApochromat 63× oil immersion objective (numerical aperture: 1.40; Leica Microsystems). Scanning settings of FRAP microscopy were as follows: unidirectional scanning at 400 Hz, 2x optical zoom, and image format 512 × 256. Single scans (n = 30) were imaged with 4% laser power intensity (pre-bleaching) at 640 nm, followed by bleaching of the region of interest (ROI of 4 µm^2^ in area) 10 times with five laser lines (608, 616, 624, 632 and 648 nm) set to maximal power. Then, cells were imaged using the same settings as in the pre-bleach recordings, and the fluorescence signal recovery was recorded by sequential imaging scans (63 frames, for 1 min), using LAS X software. FRAP recovery curves were generated from background subtracted images and the values of fluorescence intensity at each time point were singly normalized to the initial fluorescence as described (Phair and Misteli, 2000). Curve fitting was done using a single term exponential equation with the software easyFRAP (Rapsomaniki et al., 2012). Curve fits with regression values (*R* ^2^) lower than 0.9 were excluded from the analysis.

### 2.6 GFP-EB3 assay

Neurons at day 20 of cortical differentiation were cultured in 35 mm μ-dish with glass bottom (#81156, Ibidi) pre-coated with Matrigel, and transfected with 1 μg end binding protein 3 (EB3)-GFP plasmid kindly provided by Qiang L and Baas PW, Muralidharan and Baas (2019) using Polyethylenimine (PEI) (23966, Polysciences) to visualize EB3 “comets.” Twenty-four hours after transfection, live-cell imaging was conducted at stable conditions of temperature, CO_2_ and humidity in microscope stage incubator (OkoLab). Confocal time-lapse microscopy of the EB3 transport experiments were performed on a Leica TCS -SP8X AOBS laser-scanning confocal microscope (Leica Microsystems), equipped with a white light laser (470–670 nm) source, a 405 nm laser. Images were acquired using a PlanApochromat 63× oil immersion objective (numerical aperture: 1.40; Leica Microsystems). GFP-tagged EB3 transport imaging was carried out for 2 min in XYt mode (optical section of 2 µm) using a low excitation of the single 488 nm line of the white light laser (1.4% power), a bidirectional scanning mode at 500 Hz (0.526 s/frame) of speed, 6× optical zoom, 512 × 512 of image format with a pixel resolution of 60 nm. Then, confocal GFP-tagged EB3 images were deconvolved with Hyvolution2 module (Huygens) and tracked automatically using u-track software release 2.2.0 (Applegate et al., 2011). “Comet detection” parameters were individually optimized both to avoid false positive detections and to minimize false negative detections. The minimum track length was set to three frames and we allowed comets to disappear for a maximum of two frames. All tracking parameters remained at default settings except for the followings: search radius 1–5 pixels, maximum forward angle 15°, maximum backward angle 5°. Comets directions were extracted from the “tracksFinal” structure and analysed using a costume MatLab script. Microtubule dynamics parameters were compiled from multiple individual experiments.

### 2.7 Statistical analysis

Results were referred to a minimum of three independent experiments and the data were blindly analysed and represented using mean and standard error of the mean (mean ± SEM). Significance was tested using unpaired *t-*test or ordinary one-way ANOVA for normally-distributed data, and nonparametric tests when normal distribution could not be assessed. GraphPad-Prism software (Prism 8.0.2, GraphPad Software) was used to analyze the data.

## 3 Results

### 3.1 PCDH19 affects MF polymerization in iPSC-derived cortical neurons

iPSC lines from healthy donors (Ctrl) and one iPSC line from a male patient carrying the p.Pro451Leu variant, were successfully differentiated into cortical neurons using a growth factor-based protocol (as described in Borghi et al., 2021)^.^ To unveil the MF dynamics we examined the polymerization capacity of MFs following differentiation in cortical neurons (at day 30), inducing MFs de-polymerization by cytochalasin D treatment. After drug washout, we monitored the re-polymerization status of MFs at different times (0, 15, 45, 75 min) by immunofluorescence staining of β-actin antibody (Figure 1A). The results show that MFs of the iPSC-derived neurons carrying the PCDH19 variant have slightly increased β-actin fluorescence signal and that cytochalasin D treatment has the same de-polymerizing effect on control and PCDH19-mutated neurons (as visible from the similar slope of the orange and blue lines between the points “untreated” and “0 min” in Figure 1B). Despite this, a significative difference is observed after 15 min from the agent washout between the samples (P = 0.0184, ordinary two-way ANOVA test), showing a different timing of MFs repolymerization in PCDH19-mutated neurons. Noteworthy, the β-actin fluorescence in the untreated condition and at 45 (and 75) minutes following cytochalasin D washout is (not significantly) increased in the PCDH19-mutated neurons.

### 3.2 PCDH19 affects MT polymerization in iPSC-derived cortical neurons

**FIGURE 2:**
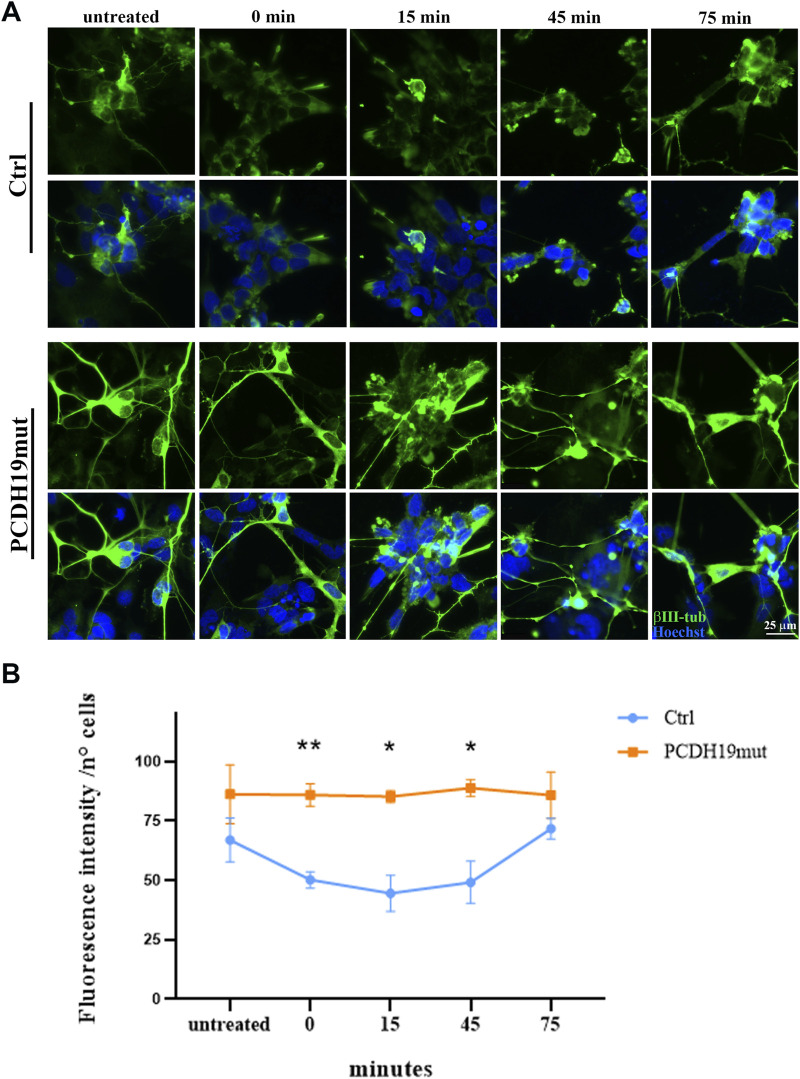
Impairment of microtubules depolymerization in PCDH19mut iPSC-derived cortical neurons. **(A)** Representative immunofluorescence analyses showing resistance to nocodazole de-polymerization of βIII-Tubulin (green) in cortical neurons derived from PCDH19mut iPSCs as compared to control cells before (untreated) and after treatment (at 0, 15, 45, and 75 min following agent washout). Nuclei are marked with Hoechst33342 (blue). Scale bar = 25 μm. **(B)** Quantification of βΙΙΙ-tubulin fluorescent signal intensity. A minimum of n = 3 and 9 images were quantized per experimental condition. Data are represented as mean ± SEM of three independent experiments and the number of cells analysed is between 1,014 and 1,097 per experimental condition. Significance was tested using ordinary two-way ANOVA (parametric tests). *p ≤ 0.05; **p ≤ 0.005.

To study MT dynamics, we induced MTs depolymerization by nocodazole treatment and the re-polymerization process was analyzed by immunofluorescence staining of βΙΙΙ-tubulin on cortical neurons fixed before the nocodazole treatment (untreated) and at 0, 15, 45 and 75 min after its removal. Importantly, the fluorescent images and the quantification analysis of βΙΙΙ-tubulin show that the MTs of PCDH19-mutated cortical neurons are resistant to nocodazole de-polymerization when compared to control cortical neurons (Figures 2A, B). In particular, following nocodazole treatment (at 0, 15 and 45 min after washout) the fluorescence intensity is significantly different among control and PCDH19-mutated neurons (**p = 0.002 at 0 min; *p = 0.0203 at 15 min; *p = 0.0405 at 45 min).

### 3.3 MTs are more stable in PCDH19 mutated cortical neurons

**FIGURE 3:**
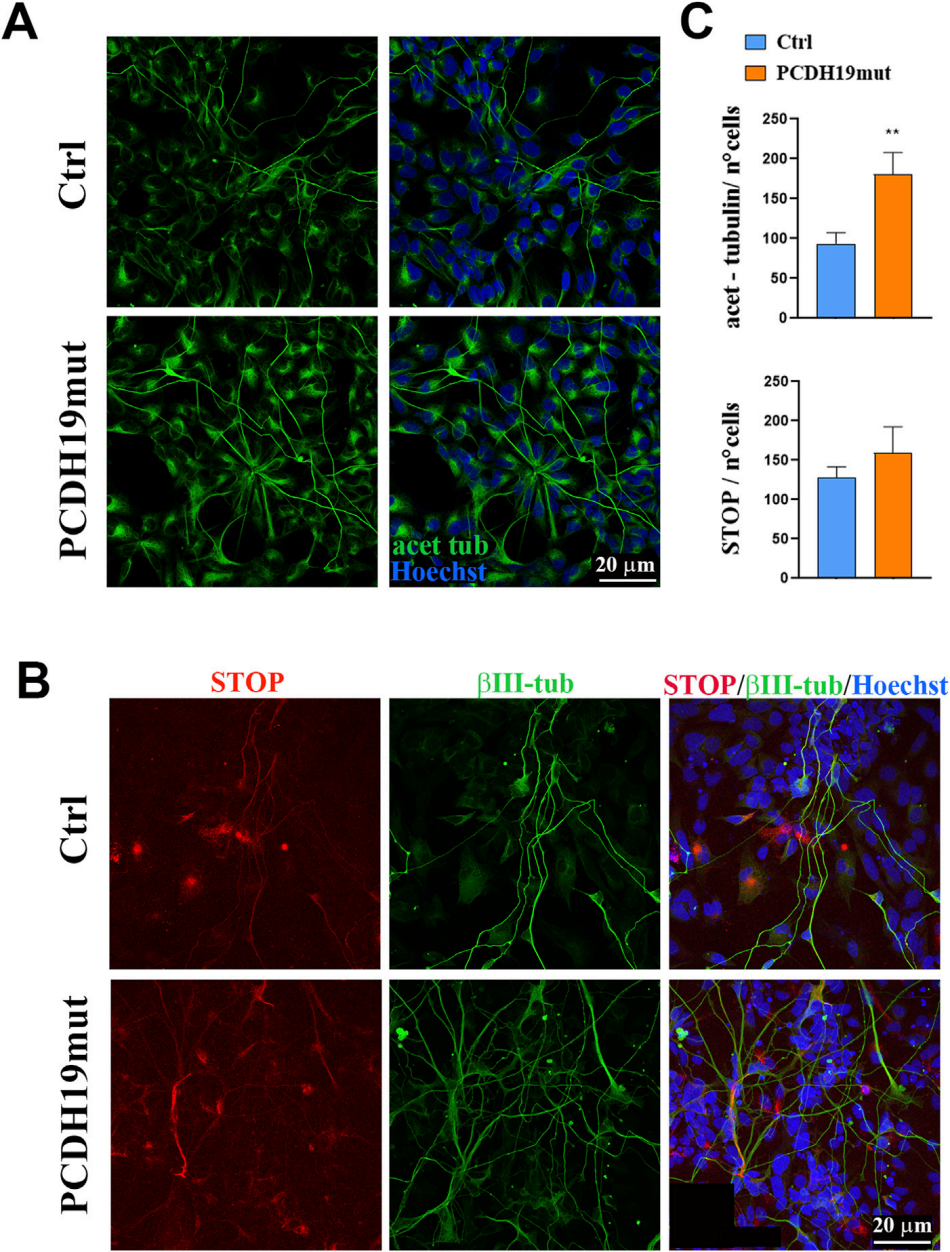
Microtubule stability is increased in PCDH19mut iPSC-derived neurons. Immunostaining to visualize cortical neuron stability. **(A)** Representative immunofluorescence images for acetylated-tubulin in control (Ctrl) and PCDH19mut cortical neurons. Scale bar = 20 μm. **(B)** Confocal micrographs of immunofluorescence for STOP and βΙΙΙ-Tubulin on Ctrl and PCDH19mut cortical neurons. Scale bar = 20 μm. **(C)** Bar graph depicting quantification of fluorescence signal of acetylated-tubulin and STOP shows that their levels are increased in patient’s derived neuronal culture compared to control. The number of cells analysed for acet-tubulin quantification analyses is 758 for control and 725 for PCDH19-mutated cells; the number of cells analysed for STOP quantification analyses is 1,005 for control and 1,020 for PCDH19-mutated cells. Data are normalized to the number of cells and presented as the mean ± SEM, n = 3 (**p ≤ 0.005).

To understand if the PCDH19 variant p.Pro451Leu leads to increased MTs stability, we performed immunofluorescence assays for known markers of stable MTs, as acetylated-tubulin and stable-tubule-only-polypeptide (STOP) (Figures 3A, B). Quantification of fluorescence intensity provided evidence of significantly increased levels of acetylated-tubulin in PCDH19mut cortical neurons when compared to control ones (**p = 0.0076, unpaired *t*-test, mean ± SEM, n = 9 images, N = 3 independent experiments), whereas the levels of STOP were slightly increased though such difference did not reach statistical significance (*p* = 0.3600, unpaired *t*-test, mean ± SEM, n = 9 images, N = 3 independent experiments) (Figure 3C).

### 3.4 FRAP analyses show impairments of PCDH19mut neurites’ terminals

**FIGURE 4:**
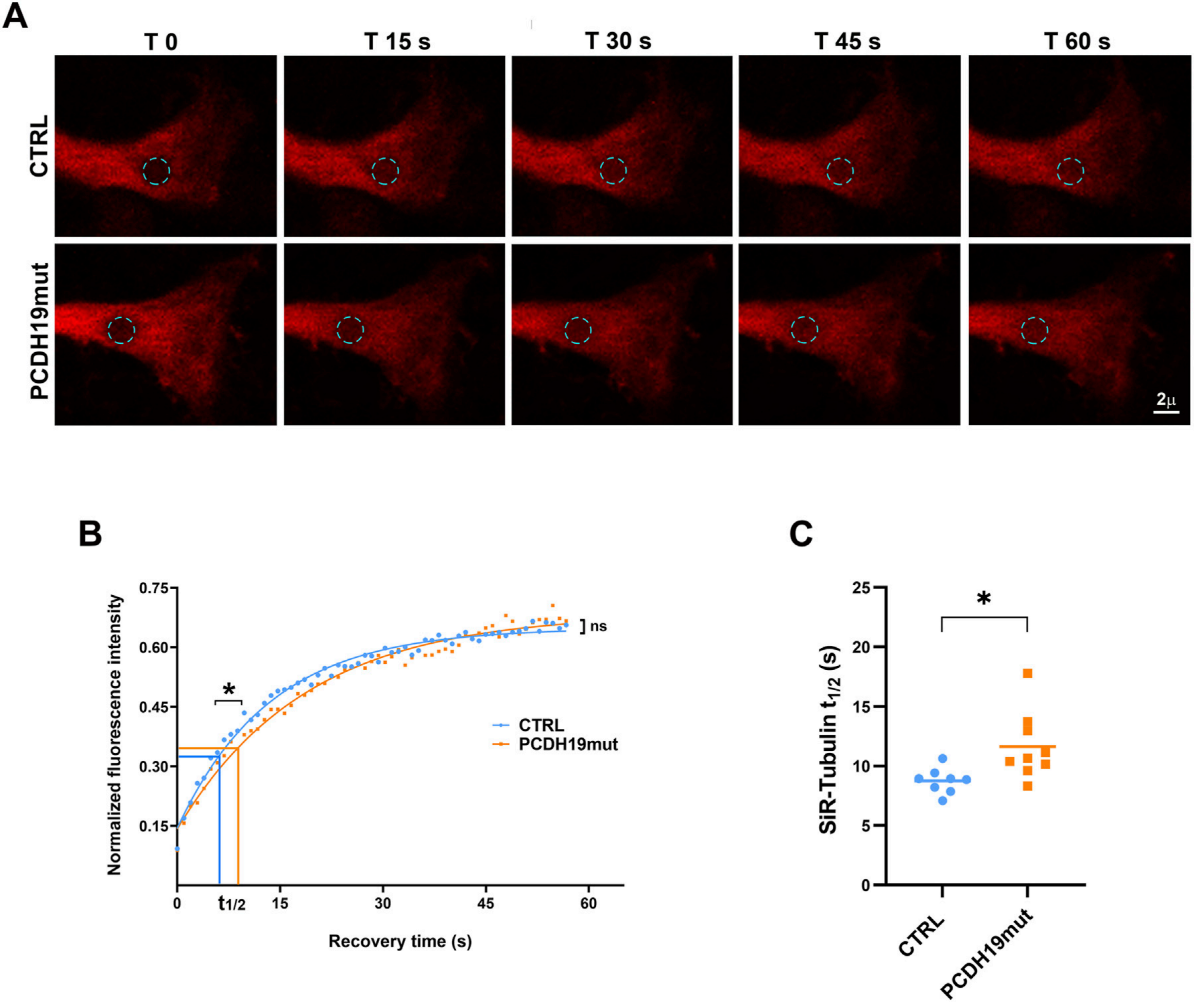
FRAP analysis of distal neurites ends reveals different MT dynamics between control and PCDH19mut cortical neurites. **(A)** Representative images of FRAP experiments carried out at the distal ends of control (Ctrl) and PCDH19mut neurites by recovery imaging of the SiR-Tubulin fluorescence probe in the bleaching circular area (cyan dotted circle) for 60 s. **(B)** FRAP recovery curves of the average fit of data (continuous lines) showed significant difference (*p ≤ 0.05) in the half-maximal recovery time values (t1/2) suggesting a slower recovery kinetics, although a continuous recovery was observed in the second phase of the curve (ns: not significant). **(C)** Pooled quantitative data of the time to reach half-maximal recovery of fluorescence (t_1/2_) highlighted a slower recovery of PCDH19mut (*p ≤ 0.05) compared to control neurites: Data are mean ± SEM of n ≥ 8 neurites of control and PCDH19mut in three independent experiments.

To further investigate the details of MTs polymerization on living cells, we performed FRAP experiments using a permeant specific microtubule probe, the SiR-tubulin, to study the tubulin dynamics in control and PCDH19mut neurons, by monitoring the fluorescence recovery rate in a bleached region using time-lapse confocal microscopy. To this purpose, after a pre-bleaching imaging step, an irreversibly bleaching was induced in a specific circular area of 4 μm^2^ at the distal ends of living cortical neurites, then fluorescence recovery curves deriving by unbleached molecules moving into the bleached region were recorded (Figure 4A). In our hands, FRAP recovery curves of SiR-tubulin were best fitted by a mono-exponential equation. This photobleaching method enabled us to identify a significant difference between control and PCDH19mut neurons in the first phase of the recovery curve where the half-maximal recovery time (t1/2) of patient’s samples (11.64 ± 0.94 *versus* 8.75 ± 0.38 of control samples; *p* = 0.0159) displayed slower dynamics suggestive of a reduced diffusion rate of tubulin molecules (Figures 4B, C). In the second phase, the recovery kinetics appeared continuous and reached average values comparable to control ones (Figure 4B). Consequently, no significant differences were found in the relative mobile fraction between PCDH19-mutated (0.67 ± 0.02) and control (0.64 ± 0.01) samples (data not shown).

### 3.5 MT dynamics is impaired in PCDH19 mutated cortical neurons

With the aim to investigate further on MT dynamics, we performed GFP-EB3 imaging of cultured control and PCDH19mut cortical neurons to visualize MT growth events by quantitative analyses of density, orientation and speed of EB3 comets using time-lapse confocal microscopy. EB3 is one of the microtubule plus-end tracking proteins (+TIPs), a category of protein associated with the plus ends of elongating MTs with the function of modulate their dynamics and their ability to control cell polarity (van de Willige et al., 2016; Poobalasingam et al., 2022).

**FIGURE 5:**
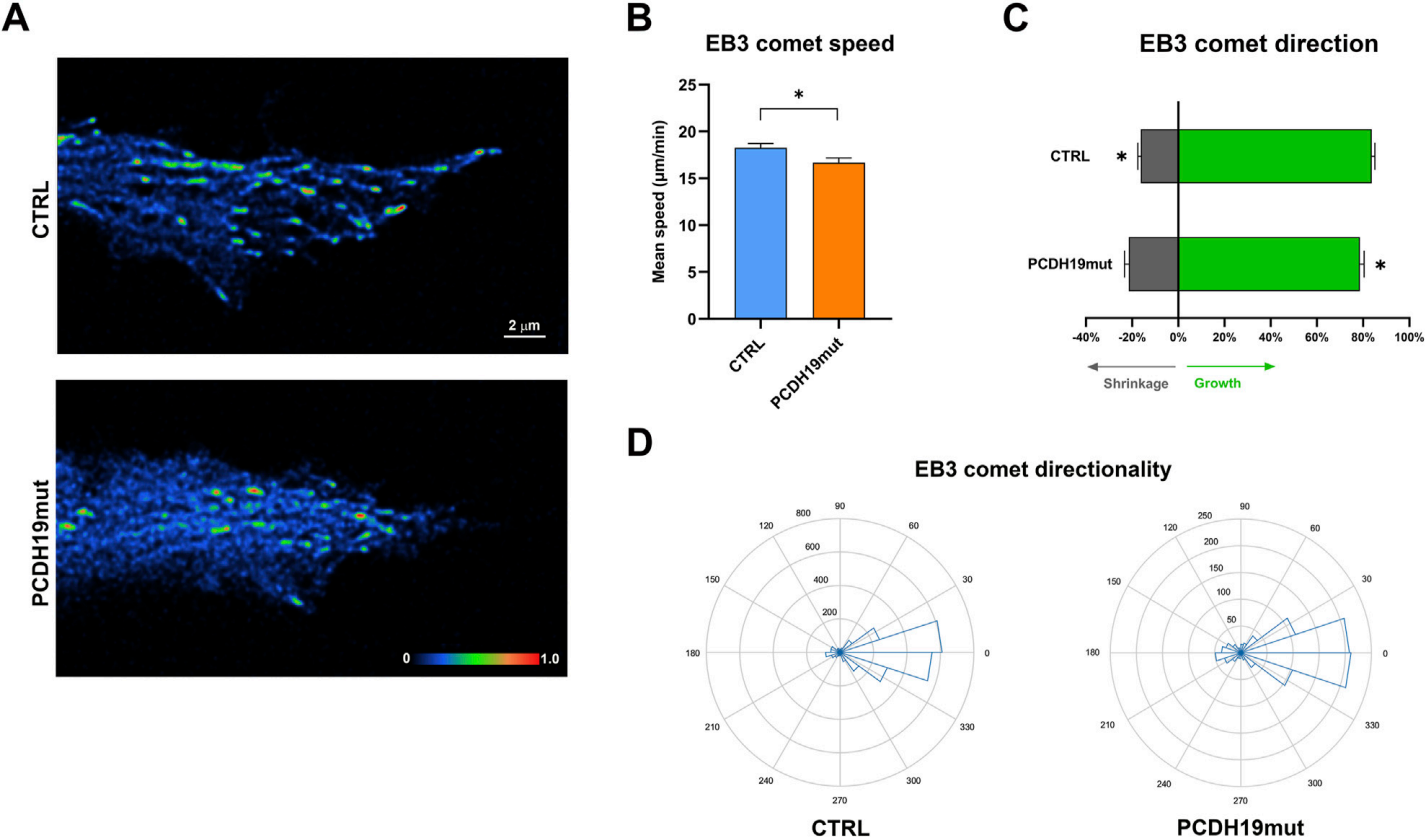
MT dynamics assayed by time-lapse imaging of GFP-EB3 comets in control (Ctrl) and PCDH19mut cortical neurons. **(A)** Representative time-lapse images of EB3 comets distribution at the distal ends of cortical neurites from Ctrl (upper panel) and PCDH19mut (lower panel) samples. Comet intensities were represented by “Physics” LUT color-coded scale bar using Fiji software (Image J). **(B)** Bar graph of average speed of EB3 comets measured at the ends of control and PCDH19mut cortical neuritis (*p < 0.05). **(C)** Bar graph shows Mean frequency of EB3 comet orientation in PCDH19mut cortical neurites resulted more predominant in the retrograde (grey) than anterograde (green) direction compared to control cells (*p ≤ 0.05). **(D)** Polar histogram plots showing the EB3 comet directionalities in relation to neurite orientation (anterograde to right, retrograde to left) in control and PCDH19mut cortical neurons. Data are mean ± SEM of n ≥ 15 neurites of Ctrl and PCDH19mut, respectively, in three independent experiments.

We focused our analysis at the distal end of cortical neurites following the displacements of GFP-EB3 comets by consecutive frames for 2 min. Then, after deconvolution, tracking image sequences were analyzed using u-track software (Applegate et al., 2011). The analyses of GFP-EB3 trajectories (Figure 5A; Supplementary Video S1) highlighted a reduced mean speed value in PCDH19mut neurites (16.68 ± 0.50 μm/min) suggestive of lower microtubule dynamics compared with control samples (18.26 ± 0.45 μm/min; *p* = 0.0279, Figure 5B). In addition, PCDH19-mutated cortical neurites showed some differences in directionality and orientation of GFP-EB3 comets with more predominant transport events in the retrograde (18.26% ± 1.82%) rather than anterograde (78.54% ± 1.82%) direction compared to control cells (retrograde: 16.68% ± 1.26%; anterograde: 83.74% ± 1.3%, *p = 0.0203; Figure 5C). The graphical representation of comet directionalities in relation to neurite orientation, displayed in radial polar histogram plots (anterograde to right, retrograde to left) well reflected these results in control and PCDH19mut cortical neurons (Figure 5D).

## 4 Discussion

Recent studies consistently documented that PCDH19 dysfunction leads to impaired neuronal development and particularly neurites’ length and mitotic spindle orientation (Pederick et al., 2016; Homan et al., 2018; Borghi et al., 2021). Since this process is strictly associated to cytoskeleton functionality (Compagnucci et al., 2016a), we decided to further characterize neuronal cytoskeletal dynamics.

Both MFs and MTs are cytoskeletal elements finely modulated during various neurodevelopmental processes, including neuronal migration, axon outgrowth and function, dendritic spine formation, plasticity and establishment of neural connectivity (Sheng and Hoogenraad, 2007; Witte and Bradke, 2008; Gu et al., 2008; Jaworski et al., 2009; Marín et al., 2010; Kevenaar and Hoogenraad, 2015; Spence and Soderling, 2015; Pchitskaya et al., 2022). Dysfunction of proteins involved in actin dynamics and organization (e.g., Rho-associated protein kinase), which are able to regulate neurite outgrowth, growth cone stability and axon elongation, have previously been linked to human neuronal pathology by studies of neuronal cultures obtained from patient-derived iPSCs (Compagnucci et al., 2016b).

Previous studies reported that the δ2 non-clustered protocadherins, including PCDH19, promote dynamic cellular processes as cell motility (Nakao et al., 2008; Biswas et al., 2010; Hayashi et al., 2014; Hayashi and Takeichi, 2015) and, in fact, loss of pcdh19 causes cell movement defects during anterior neural plate development in zebrafish embryos (Biswas et al., 2010) and altered neuronal migration of rat hippocampal neurons during brain development (Bassani et al., 2018).

Additionally, altered mitotic spindle formation (Borghi et al., 2021) and impaired dendrite morphology (Bassani et al., 2018), support the hypothesis that PCDH19 has an impact on MF and MT organization and dynamics. Importantly, two independent studies based on zebrafish and mouse models, show that pcdh19 interacts with proteins involved in actin and microtubule regulation (Emond et al., 2021; De Nys et al., 2024). For this reason, we focused on investigating the cytoskeletal organization to understand the cellular aspects underlying PCDH19-CE. Specifically, we investigated the involvement of PCDH19 in MF and MT dynamics using iPSC-derived cortical neurons obtained from a mosaic male patient with PCDH19-CE (Borghi et al., 2021), carrying the pathogenic variant c.1352C>T (p.Pro451Leu), which is a missense variant (as the most frequently reported type of variant, 45.4%) on exon 1, where the majority of the described variants were observed (86.7%) (Kolc et al., 2019).

The data obtained in the present study demonstrate that PCDH19 dysfunction leads to altered a different re-polymerization of actin filaments following cytochalasin D treatment, thus affecting MF dynamics in mutated iPSC-derived cortical neurons (Figure 1). This result is supported by the evidence that, as already demonstrated for other δ2-protocadherins (PCDH10, PCDH17, PCDH18) (Nakao et al., 2008; Hoshina et al., 2013; Biswas et al., 2014; Hayashi et al., 2014; Hayashi and Takeichi, 2015; Light and Jontes, 2017), the conserved cytoplasmic region of PCDH19 contains a key motif, termed WRC interacting receptor sequence (WIRS), which is responsible for binding to WRC, through which it regulates actin dynamics (Nakao et al., 2008; Hayashi et al., 2014; Chen et al., 2014; Tai et al., 2010; Pancho et al., 2020; Emond et al., 2021). Since in neurons WRC proteins are localized at the growth cones and regulate axon growth (Yokota et al., 2007) through dynamically re-modelling of actin cytoskeleton, it is possible that PCDH19, through its interaction with the WRC, regulates actin nucleation activity, which is important for proper dendritic arborization in developing neurons (Spence and Soderling, 2015), as well as other Pcdhs (Nakao et al., 2008; Biswas, 2014; Hayashi et al., 2014; Hayashi and Takeichi, 2015; Fan et al., 2018). Alterations of MF organization have been observed for other protocadherins, as for PCDH17 (another member of the δ2 subfamily associated to DEE9), which regulates axon extension by engaging actin polymerization regulators to inter-axonal contact sites, converting these sites into motile structures (Hayashi et al., 2014).

Other studies linking actin cytoskeleton and epilepsy include those on the epileptic brain, where it was observed an altered dynamics of actin cytoskeleton following status epilepticus (Yang et al., 2019; Gambino et al., 2022). Importantly, epileptic encephalopathy early infantile 65 (EIEE65, caused by dysfunctional CYFIP2, a protein belonging to WRC) is characterized by early onset of intractable seizures and severe psychomotor developmental delay (Nakashima et al., 2018), which are clinical features in common with PCDH19-CE (also known as epileptic encephalopathy, early infantile 9, EIEE9). This similarity strongly supports that PCDH19 is involved in MF dynamics.

A role for PCDH19 also in MT organization is supported by recent studies that highlighted the involvement of PCDH19 in cell division processes. In fact, during mitosis, PCDH19 localizes at the poles of the mitotic spindle, close to the gamma-tubulin (Compagnucci et al., 2015; Borghi et al., 2021), where it interacts with neural precursor cell expressed, developmentally downregulated 1 (NEDD1) (Emond et al., 2021), a protein that is localized at the centrosome and that binds to the components of the gamma-tubulin ring complex (Manning and Kumar, 2007). Interestingly, iPSCs with mutated PCDH19 present centrosome hyper-amplification (Borghi et al., 2021), suggesting that PCDH19 dysfunction leads to an altered mitotic spindle formation followed by premature differentiation. Therefore, in addition to the effect of PCDH19 dysfunction on the MFs, we decided to also characterize the MTs and we obtained an impairment of MT dynamics in patient-derived cortical neurons. In fact, MT de-polymerization, induced with nocodazole treatment by sequestering tubulin dimers, is not observed in PCDH19 mutated culture (Figure 2), suggesting an impairment in the dynamics of these cytoskeletal components due to more stable MTs. Increased stability of MTs is confirmed by increased levels of acetylated α tubulin and STOP (Figures 3A–C), which are both associated with the stable portion of MTs (Piperno et al., 1987; Cuveillier et al., 2021; Carmona et al., 2023). Interestingly, together with increased neurites’ length in PCDH19-mutated neuronal cells (Borghi et al., 2021), a significant increase in neuronal branching is reported in PCDH19 KO cells (Mincheva-Tasheva et al., 2021). Further studies are needed to unveil how these data correlate with increased MTs stability and why PCDH19 protein impairment leads to MTs alteration.

To investigate the growing end of neurites, where the MTs dynamic is crucial for axonal growth and establishment of synapses and network functionality, we assessed the impact of PCDH19 in MT polymerization capacity by performing FRAP experiments of SiR-Tubulin stained cortical neurons at the distal ends of neurites (Figure 4A). We used SiR-Tubulin probe, a known bright, photostable and permeable protein based on silicon-rhodamine (SiR) dye (Lukinavičius et al., 2013) and the microtubule binding drug Docetaxel. Its brightness allows lower exposure time or laser power during imaging, whereas its near far-red adsorption and emission minimize cytophototoxicity (Lukinavičius et al., 2014) and unwanted autofluorescence background allowing to obtain a high signal-to-noise ratio. These properties translate into the best performance of live cell imaging aimed at quantitative studies of biological phenomena. The data obtained show that the time to reach the half-maximal of fluorescence recovery (t 1/2) is higher in PCDH19mut neurons when compared to control ones (Figures 4B, C), and the recovery curve shape suggested a typical diffusion rate of a protein that transiently interacts with relatively immobile cellular structures such as cytoskeleton (Phair and Misteli, 2001; Lippincott-Schwartz et al., 2003). Our results pointed out crucial aspects of PCDH19mut cortical neurons, where MTs are characterized by a slower growth.

To deepen the analyses of MT dynamics, we tracked the plus end of MTs using EB3 comets on iPSC-derived cortical neurons demonstrating that EB3 comets speed in PCDH19mut neurons is decreased (Figures 5A, B and Supplementary Video S1) and that EB3 comets direction is less anterograde and more retrograde compared to control iPSC-derived neurons (Figures 5C, D). These data confirm that PCDH19mut iPSC-derived neurons are characterized by decreased MT polymerization rate, and suggest more stable MTs, in agreement with a decreased speed of EB3 comets in PCDH19mut iPSC-derived neurons (Jaworski et al., 2009; Qiang et al., 2018).

Despite PCDH19-CE is manifested in heterozygous females or mosaic males, where two cell populations carrying two different PCDH19 proteins co-exist (one encoded by the wild type gene and the other by the *PCDH19* variant), we decided to focus on control and PCDH19-mutated neuronal cultures only, avoiding mixed control/PCDH19mut cultures for two reasons. The first reason is the necessity to characterize the cell autonomous features of PCDH19-mutated neurons, the second is the technical difficulty in visualizing and knowing the precise percentage and localization of PCDH19-mutated *versus* wild type neuronal cells without stressful experimental manipulations (i.e., transient GFP transfection or constitutive transduction of viral vectors) in mixed cell cultures. Additionally, in several animal models of Pcdh19 loss of function (i.e., mouse, zebrafish and *Xenopus*), Pcdh19 loss causes hyperexcitability and autism-like behaviors also in males (Lim et al., 2019; Park et al., 2024; Robens et al., 2022). These data suggest that mutated PCDH19 contributes to disease phenotype through mechanisms other than cellular interference in mosaic condition, and this is in agreement with clinical studies which have identified human males with ASD (autism spectrum disorder) carrying variants in *PCDH19* gene (Piton et al., 2011; van Harssel et al., 2013; Chouery et al., 2023).

In conclusion, despite in our PCDH19-CE *in vitro* human model we demonstrate impairment of MT dynamics/stability, it remains to be understood how PCDH19 dysfunction causes these alterations. Therefore, further studies are necessary to deeply unveil the mechanisms by which PCDH19 impairment affects MTs in PCDH19mut neurons.

## Data Availability

The data presented in this study have been deposited in Zenodo with the following doi: 10.5281/zenodo.14505987.

## Supplementary material

SUPPLEMENTARY VIDEO S1 Visualization of GFP-EB3 comets’ movements in transfected control (Ctrl) and PCDH19mut cortical neurons. The videos show the GFP-EB3 movement in neurons obtained differentiating control- (on the left) and patient’s-derived iPSCs (on the right). Cortical neurons were transfected with 1 μg GFP-EB3 plasmid and maintained in neuronal culture medium at 37°C. The dynamics of the comets (contained in the 2 μm thick optical section) were documented by confocal microscopy using a low excitation of the single 488 nm line of the white light laser and detected for a time recording of 2 min.
